# Kaposi sarcoma in a female patient with multiple sclerosis: a case report

**DOI:** 10.1186/s13256-025-05683-4

**Published:** 2025-11-21

**Authors:** Ahmad Al-Bitar, Ahmad Alkhaledi, Malek Alhamedi

**Affiliations:** 1 Faculty of Medicine, Damascus University, Damascus, Syria; 2 Faculty of Medicine, Al-Bairouni University Hospital, Damascus University, Damascus, Syria; 3 Al-Bairouni University Hospital, Head of Sarcoma Unit, Damascus, Syria

**Keywords:** Kaposi sarcoma, Multiple sclerosis, Paclitaxel, Case report

## Abstract

**Background:**

Kaposi sarcoma is a vascular neoplasm linked to human herpes virus 8 and presents in four main variants. The development of Kaposi sarcoma, particularly the iatrogenic form, in younger women with autoimmune conditions such as multiple sclerosis is exceedingly rare and presents unique diagnostic and therapeutic challenges.

**Case presentation:**

We report the case of a 48-year-old Arab woman with an 18-year history of multiple sclerosis managed with corticosteroids, who developed cutaneous iatrogenic Kaposi sarcoma on her lower extremities. The diagnosis was delayed for 2 years as the lesions were initially misattributed to her underlying multiple sclerosis. A skin biopsy eventually confirmed Kaposi sarcoma, and staging revealed localized cutaneous disease with no visceral involvement. The patient was treated with weekly paclitaxel, showing a favorable response. Due to conflict-related logistical issues, treatment was temporarily switched to vincristine and vinorelbine, which resulted in disease progression and adverse effects. Paclitaxel was successfully reintroduced, leading to sustained clinical improvement, and 2 years after completing therapy, the patient remains in good health with no evidence of Kaposi sarcoma recurrence or multiple sclerosis exacerbation.

**Conclusion:**

This case expands the known clinical and demographic spectrum of Kaposi sarcoma, underscoring the importance of maintaining a high index of suspicion in atypical patient populations to prevent diagnostic delays. The co-occurrence of multiple sclerosis and Kaposi sarcoma underscores the importance of recognizing iatrogenic Kaposi sarcoma in patients receiving immunosuppressive therapy and calls for further research into the complex intersection of autoimmune disorders and iatrogenic treatment-related immune dysregulation in oncogenesis. This report underscores the importance of individualized, persistent therapeutic strategies in managing Kaposi sarcoma.

**Supplementary Information:**

The online version contains supplementary material available at 10.1186/s13256-025-05683-4.

## Background

Kaposi sarcoma (KS) is a multicentric angioproliferative neoplasm of endothelial cell origin, fundamentally linked to infection with human herpes virus-8 (HHV-8). [1] First described by Moritz Kaposi in 1872, KS is now classified into four major clinico-epidemiological variants:

- **Classic KS** typically presents as indolent, purple-to-brownish plaques and nodules on the lower extremities of elderly men of Mediterranean or Eastern European descent.
- **Endemic KS** is found in sub-Saharan Africa, can be more aggressive, and may affect children and younger adults.
- **Epidemic KS** is the well-known acquired immunodeficiency syndrome (AIDS)-associated variant, which was a common opportunistic malignancy before the advent of effective antiretroviral therapy.
- **Iatrogenic KS** occurs in immunocompromised individuals, most notably solid-organ transplant recipients and patients receiving long-term immunosuppressive therapy for autoimmune diseases and other conditions [1, 2].

While KS is often associated with profound immunosuppression, its occurrence in human immunodeficiency virus (HIV)-negative individuals treated for autoimmune disorders is an area of growing recognition. Multiple sclerosis (MS) is a chronic autoimmune demyelinating disease of the central nervous system that involves inherent immune dysregulation and is often managed with immunomodulatory treatments, establishing a potential risk context for complications such as KS [3].

In this report, we showcase a 48-year-old woman who has been living with multiple sclerosis for 18 years. Recently, she was diagnosed with Kaposi sarcoma and underwent chemotherapy, leading to a successful response without affecting or inducing multiple sclerosis.

## Case presentation

**Fig. 1:**
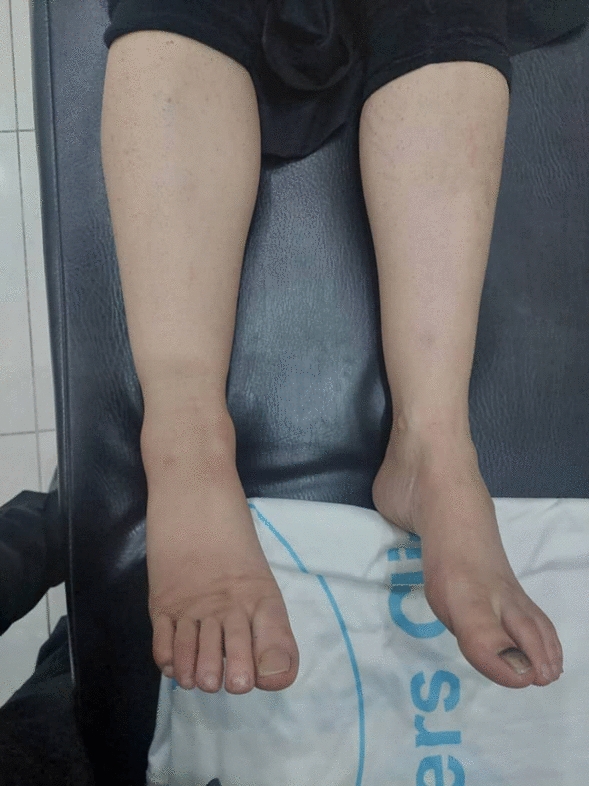
Lesions disappeared totally after treatment

A 48-year-old Arab woman presented with a purple rash on the dorsal and palmar aspects of her feet, as well as on her shins, which had first appeared 2 years prior (Fig. 1). At the time, she did not seek medical attention, attributing the lesions to her history of multiple sclerosis (MS) or possible treatment-related side effects. Her 18-year history of multiple sclerosis was managed with corticosteroids. Over the following 2 years, the lesions progressively enlarged. Skin biopsy was eventually performed, confirming the diagnosis of Kaposi’s sarcoma.

Following the histopathological confirmation of Kaposi sarcoma, a comprehensive workup was performed to rule out underlying immunosuppression. Serological testing for human immunodeficiency virus (HIV-1/2) was negative. Further immunological screening revealed no other evidence of a primary or acquired immunodeficiency.

**Fig. 2:**
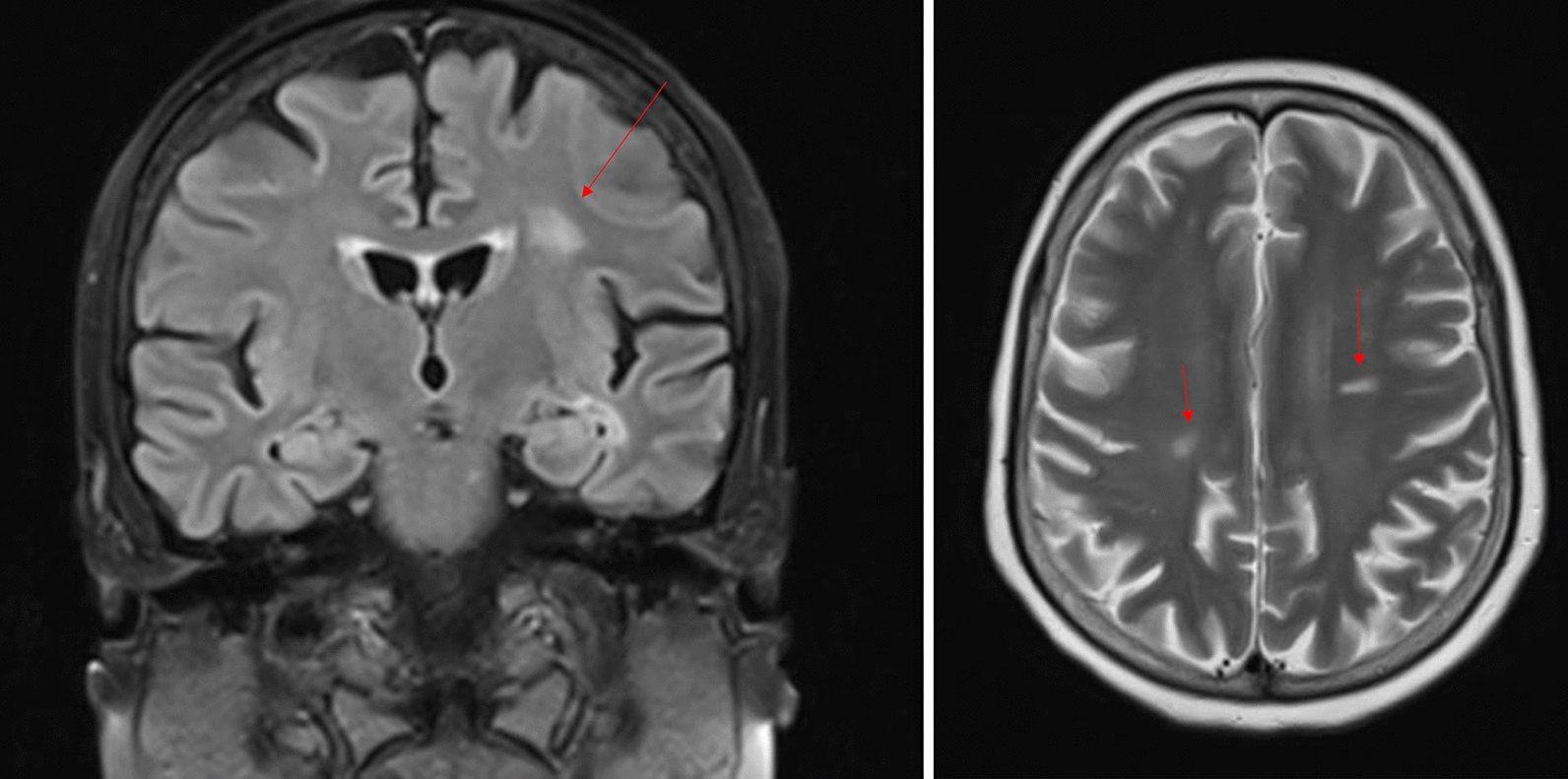
Brain MRI showing demyelinated subcortical plaques consistent with multiple sclerosis. Coronal (left) and axial (right) FLAIR-weighted images demonstrate multiple hyperintense lesions (red arrows) within the periventricular and subcortical white matter, characteristic of demyelinating plaques. No evidence of intracranial metastasis was detected

For staging, magnetic resonance imaging (MRI) of the brain was conducted to rule out metastasis. Although no metastases were detected, the MRI revealed demyelinated subcortical plaques in the cerebellum (Fig. 2) , consistent with her known MS. Multislice computed tomography (MSCT) of the chest, abdomen, and pelvis showed no evidence of distant metastasis.

In light of the diagnosis of iatrogenic Kaposi sarcoma, the suspected causative agent—systemic corticosteroids—was discontinued prior to initiating chemotherapy. The patient’s multiple sclerosis had been managed with corticosteroids for 18 years, but she remained off all immunosuppressive therapy from the start of her Kaposi sarcoma treatment onward. Systemic therapy for KS was indicated due to the progressive and symptomatic nature of the lesions. While pegylated liposomal doxorubicin is often a first-line agent, it was unavailable in our region at the time of treatment. Therefore, the patient was initiated on weekly paclitaxel.

**Fig. 3:**
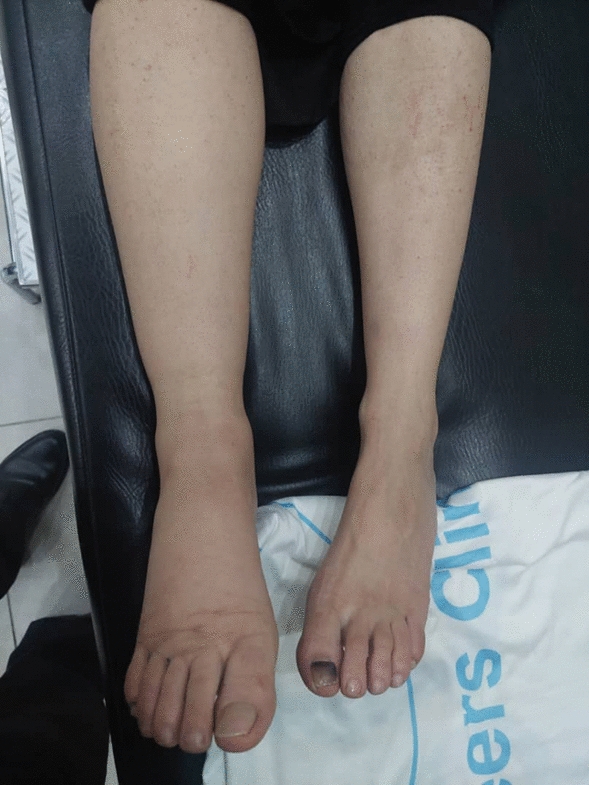
Histology slides showing spindle-shaped hypercellular tumor with an intermediate nuclear atypia rate. Immunohistochemically showed positivity for cluster of differentiation 34 and calretinin in the tumor cells

The patient was initiated on weekly paclitaxel 100 mg, with a favorable clinical response observed after 2 months of treatment (Fig. 3). However, due to conflict-related circumstances, she was switched to vincristine, which was associated with significant adverse effects, including constipation and a general decline in her health, accompanied by disease progression. Subsequently, treatment was changed to oral vinorelbine, which also failed to control the disease. Given the lack of response and logistical challenges, paclitaxel was resumed once it became available again.

Subsequently, paclitaxel was resumed at the same dose weekly, continued for a total of 60 cycles. During this time, the patient reported gradual improvement in both her cancer symptoms and MS-related complaints. She eventually discontinued treatment.

Notably, following the cessation of corticosteroids, the patient reported no clinical relapse of her multiple sclerosis over the subsequent 2-year follow-up period. Follow-up MRI showed persistent MS-related findings but no evidence of metastasis or new lesions, and 2 years after discontinuing therapy, the patient remained in good overall health.

## Discussion and conclusion

This case of Kaposi sarcoma in a 48-year-old woman with multiple sclerosis is clinically significant due to its occurrence in an atypical demographic and the diagnostic challenges it presented. Traditionally, classical KS (CKS) affects older male individuals of specific European descent, making this patient’s profile unusual and highlighting the need for a broad differential diagnosis for vascular skin lesions regardless of patient demographics.

A central challenge in this case is the clear classification of the disease. Given the patient’s 18-year history of corticosteroid therapy for MS, and the extensively documented link between immunosuppressive treatment and malignancy, this presentation is appropriately and definitively characterized as iatrogenic Kaposi Sarcoma. This classification supplants the initial consideration of a “hybrid state,” providing a clearer clinical interpretation.

The co-occurrence of KS and MS, particularly in the context of immunosuppressive MS therapies, is a growing area of concern. Our case, linked to long-term corticosteroid use, is further contextualized by reports of KS developing in patients treated with newer disease-modifying agents such as fingolimod. These cases, including one report concerning a 46-year-old man and another a 38-year-old man, both emphasize that the immunosuppression caused by MS treatment—whether corticosteroids or agents leading to prolonged lymphopenia such as fingolimod—is hypothesized to lead to the development of KS. This reduced immune surveillance, combined with an increased risk of herpes virus reactivation, is the likely mechanism that permits the progression of latent HHV-8 infection to active KS [4–7].

The pathogenesis of iatrogenic KS is rooted in the disruption of the delicate balance between the host immune system and latent HHV-8 infection. Corticosteroids, for instance, are thought to facilitate KS by inhibiting transforming growth factor-beta (TGF-β), a protein that typically suppresses endothelial cell growth. The commonality across different immunosuppressive treatments is the creation of a permissive environment for the virus. In many instances of iatrogenic KS, a primary intervention is the reduction or cessation of the offending immunosuppressive agent, which often results in disease regression [8–12].

This case also underscores the critical importance of diagnostic vigilance. The 2-year delay in diagnosis, where lesions were likely misattributed to MS-related or other changes, is a significant learning point. Clinicians must maintain a high index of suspicion and a low threshold for biopsy for any new vascular lesions in patients with chronic autoimmune conditions, particularly those receiving immunosuppressive therapy. Furthermore, vigilance includes regular, thorough physical examinations and monitoring, especially when using immunosuppressive agents.

In summary, this case of iatrogenic Kaposi sarcoma in a middle-aged woman with multiple sclerosis broadens the clinical spectrum of the disease and highlights the need for clinical awareness beyond traditional epidemiologic confines. This report contributes to the limited literature on KS in the context of MS and underscores the necessity of interdisciplinary care and individualized, persistent therapeutic strategies—such as the successful reintroduction of paclitaxel—in managing such complex clinical scenarios. The co-occurrence of these conditions raises important questions about the interplay between autoimmunity and iatrogenic-treatment-related immune dysregulation that merit further investigation.

## Supplementary Information

Additional file1

## Data Availability

Data sharing is not applicable to this article as no datasets were generated or analyzed during the current study.
